# Nasogastric tube impaction with metal-like stiffening requiring endoscopic retrieval: Rare long-term complication with suspected drug association

**DOI:** 10.6026/973206300213211

**Published:** 2025-09-30

**Authors:** Rohith Kunnath, Satheesh A. Vasudevan, Muhammad Shebin, Tess Shajan, Vidhu Victor

**Affiliations:** 1 Department of Gastroenterology and Hepatology, PK Das Institute of Medical Sciences, Palakkad, Kerala, India; 2 Department of General medicine, Dr. Moopen's Medical college, Wayanad, Kerala, India; 3 Department of Medicine, PK Das Institute of Medical Sciences, Palakkad, Kerala, India

**Keywords:** Ryle's tube/ Nasogastric tube impaction, PVC degradation, levetiracetam, endoscopic removal, gastric reaction

## Abstract

Nasogastric (Ryle's) tubes are widely used for enteral feeding but may rarely cause complications like stiffening, degradation, and impaction. We report a case of tube impaction in a 67-year-old female with multiple comorbidities on prolonged levetiracetam therapy. Endoscopic removal revealed the tube to be unusually rigid and structurally altered. Two similar cases with long-term levetiracetam use suggest a possible association with tube degradation. Thus, we show the importance of timely tube replacement and the need for research on drug-material interactions.

## Background:

The nasogastric tube (NGT), commonly referred to as Ryle's tube, is a very useful clinical tool that is frequently used for gastric decompression, enteral feeding, medication delivery and diagnostic procedures; particularly among those with swallowing difficulties and those requiring long-term nutritional support. NGTs are thought to be safe, but can have a number of potential complications [1]. Short-term complications include malposition, aspiration, obstruction of the tube, dislodgement of the tube, and trauma to the upper aerodigestive tract. Long-term placement may present less common but clinically significant complications [2]. It is common practice to routinely replace NGTs to try to mitigate the complications of long-term indwelling tubes; nevertheless, there are very rare and infrequent events such as stiffer tubes, natural degradation of latex, or impaction that can still present significant clinical challenges [3]. Long-term indwelling nasogastric tubes have material adaptations and complications due to sustained exposure to gastric secretions (with varying pH levels), mechanical stress, and interactions with drug or medications [4]. More specifically, Ryle's tubes (in the case of prolonged exposure), fashioned out of polyvinyl chloride (PVC), pose risks of chemical degradation under the conditions described above, though the mechanism of which is not completely understood [5]. The majority of vascular changes are typically only mild but severe stiffening (which may be referred to as "metal like") that results in impaction, may require additional interventions (endoscopic retrieval). There may be multiple contributory factors, such as the inherent nature of the tube polymer, the environment of the GI tract, and medications that may change gastric acidity or chemically react with the polymer material. Systematic evidence of the relationship between medications and NGT stiffening is weak, and available reports are mostly anecdotal. Therefore, it is of interest to describe a rare long-term complication of nasogastric tube use, characterized by metal-like stiffening and impaction requiring endoscopic retrieval, with a suspected association to concomitant drug therapy.

## Case report:

A 67-year-old woman was referred to the emergency department (ED) with complaints of being unable to feed through a nasogastric (NG) tube. There was a past medical history of intracerebral hemorrhage (ICH), Parkinson's disease, diabetes mellitus and hypertension. The patient was also taking long term levetiracetam for seizure prophylaxis, Human Mixtard for diabetes and Arkamin 0.1 mg for hypertension. On presentation the patient was hemodynamically stable. Routine general and physical examination were unremarkable. The systemic examination demonstrated reduced power in the right upper and lower extremities consistent with prior cerebrovascular accident (CVA) and difficulty with speaking. The NG tube had a blockage on first assessment which limited enteral feeding. The patient was treated with supportive care which included intravenous fluids and proton pump inhibitors. It was decided that Ryle's tube should be replaced and so attempts were made to manually remove the denoted tube but were unsuccessful and the patient was obviously anxious and worried. The tube remained in situ notwithstanding pulling on the tube. An ENT consult was requested, during which, an ENT team evaluated the patient, finding Ryle's tube likely impacted in the esophagus. Due to the concern for lower esophageal impaction, the ENT team appropriately referred the case to gastroenterology for further evaluation and management. The gastroenterology team attempted manual retrieval; however the tube was firmly impacted. The attempt to remove the tube was unsuccessful; therefore the patient was scheduled for esophagogastroduodenoscopy (OGD scopy) under sedation, to allow for endoscopic retrieval. The patient was sedated and carefully monitored according to sedation protocols, for OGD scopy with the aim of assessing the level of impaction and facilitating the removed the Ryle's tube. The impactation was in the esophagus, where evidence of mucosal erosions was found. The Ryle's tube was kinked, and buried in the esophageal mucosa, making retrieval more difficult. Furthermore, the tube was extremely stiff, which compromised standard cutting tools. The endoscope May change insertions, as a snare and forceps, were inserted through the endoscope and attempts to un- kinked the Ryle's tube were made. The extreme thickness and rigidity of the impacted tube precluded further unsuccessful attempts to un kink it. Consequently, a snare was passed through the endoscope and the kinked Ryle's tube was firmly attached to the endoscope. The tube was withdrawn with the endoscope using controlled traction. Upon retrieval, the tube was extremely rigid, exhibiting stiffness similar to metal, and this raised concern about chemical degradation caused by prolonged medication exposure. After the procedure, a plain chest X-ray (PA view) was performed to exclude perforation. After it was confirmed that there was no perforation, a new Ryle's tube was inserted, and enteral feeding was recommenced without further incident. Two more patients presented with Ryle's tube impaction a few months later, and their Ryle's tubes were retrieved with an endoscope. Both patients had prolonged levetiracetam therapy for seizure prophylaxis prior to the impaction. In both cases, they needed OGD scopy for retrieval as manual removal was unsuccessful. These cases raise questions about a possible association with long-term levetiracetam therapy and PVC degradation.

## Endoscopic findings and procedure:

The Ryle's tube was impacted within the esophagus, causing mucosal erosions. The tube was kinked and embedded in the esophageal mucosa, complicating removal. Additionally, the tube exhibited significant stiffening, making standard cutting tools ineffective. A snare and forceps were introduced through the endoscope, and attempts were made to straighten the Ryle's tube. However, due to the tube's extreme thickness and rigidity, correcting the kinking was unsuccessful. As a result, a snare was passed through the endoscope, and the kinked Ryle's tube was tightly secured to the endoscope. The tube was then removed along with the endoscope using controlled traction. Upon removal, the tube was found to be extremely rigid, exhibiting metal-like stiffness, raising suspicion of chemical degradation secondary to long-term medication exposure. Following the procedure, a plain chest X-ray (PA view) was performed to rule out perforation. After confirming the absence of perforation, a new Ryle's tube was inserted, and enteral feeding was resumed without complications.

## Discussion:

Nasogastric tubes have traditionally been used for nutritional provision in stroke patients and patients with swallowing difficulties. However, after a prolonged duration, nasogastric tubes are now increasingly being replaced with percutaneous gastrostomy because of attributable complications. While it is true that nasogastric tubes are generally tolerated, they can still lead to complications in about 0.3-0.8% of cases [6]. Although the nature of chemical interaction between gastric contents and the polyvinyl chloride (PVC)-based Ryle's tube, especially with certain medications, is not well understood, it may have some influence in how quickly tubes become stiff and ultimately obstructed [7]. Although we don't know specifically how levetiracetam contributes to this process, its prominent presence amongst our affected cohort corroborates the suspicion that there may be a relationship. Prolonged exposure to gastric acidity and chemically induced changes in the rigidity of gastric tubes while they sustain a gastric burden can mark various tube destruction points. Stiffness can lead to an obstructed tube that is more difficult to remove and may require an endoscopic intervention similar to the patient in this present case report [8]. Timely replacement of all Ryle's tubes is recommended to avoid failure due to physical degradation. Historically, practice guidelines state a maximum duration of the insertion period of seven days in wide bore tubes and up to 2 months with fine bore tubes [9]. Ongoing surveillance of patients with a long-term Ryle's tube access is important due to the importance of detecting early signs of degradation of the tube and avoiding complications [10]. Future research is needed to determine if there is involvement of levetiracetam or other medications in degrading PVC, or if there are alternative tube materials or coating to mitigate the complications of PVC.

## Conclusion:

Ryle's tube impaction is uncommon but serious enough to warrant endoscopic treatment. This case underscores the need for careful monitoring in patients with long-term nasogastric tubes, such as in those who receive levetiracetam. Involvement changes should be routine and potential alternative tube materials should be considered to lessen risk. Further studies are needed to identify and define the association between medication-induced PVC degradation and the safety of patients on long-term nasogastric tubes especially in patients with anti-epileptic drug convictions.
